# Perfluorooctanoic Acid–Induced Immunomodulation in Adult C57BL/6J or C57BL/6N Female Mice

**DOI:** 10.1289/ehp.10896

**Published:** 2008-02-07

**Authors:** Jamie C. DeWitt, Carey B. Copeland, Mark J. Strynar, Robert W. Luebke

**Affiliations:** 1 Curriculum in Toxicology, University of North Carolina at Chapel Hill, Chapel Hill, North Carolina, USA; 2 Immunotoxicology Branch, Experimental Toxicology Division, National Health and Environmental Effects Research Laboratory; 3 National Exposure Research Laboratory, U.S. Environmental Protection Agency, Research Triangle Park, North Carolina, USA

**Keywords:** fluorinated compounds, immunomodulation, immunotoxicity, perfluoroalkyl acids, PFOA

## Abstract

**Background:**

Perfluorooctanoic acid (PFOA), an environmentally persistent compound of regulatory concern, has been reported to reduce antibody responses in mice at a single dose.

**Objective:**

The aim of this study was to evaluate PFOA effects on humoral and cellular immunity using standard assays for assessing immune function, and to derive dose–response data.

**Methods:**

C57BL/6J mice received 0 or 30 mg PFOA/kg/day for 10 days; half of the exposed groups were switched to vehicle and half continued on PFOA for five days. C57BL/6N mice received 0–30 mg/kg/day of PFOA in drinking water for 15 days. Mice were immunized with sheep red blood cells or sensitized to bovine serum albumin in Freund’s complete adjuvant on day 10 of exposure; immune responses were determined 1 day post-exposure.

**Results:**

We found that 30 mg PFOA/kg/day given for 10 or 15 days reduced IgM synthesis; serum collected 1 day postexposure contained 8.4 × 10^4^ or 2.7 × 10^5^ ng PFOA/mL, respectively. IgM synthesis was suppressed at exposures ≥ 3.75 mg PFOA/kg/day in a dose-dependent manner, and IgG titers were elevated at 3.75 and 7.5 mg PFOA/kg/day. Serum PFOA at 3.75 mg/kg/day was 7.4 × 10^4^ ng/mL 1 day postexposure, or 150-fold greater than the levels reported in individuals living near a PFOA production site. Using a second-degree polynomial model, we calculated a benchmark dose of 3 mg/kg/day, with a lower bound (95% confidence limit) of 1.75 mg/kg/day. Cell-mediated function was not affected.

**Conclusions:**

IgM antibodies were suppressed after PFOA exposure. The margin of exposure for reduced IgM antibody synthesis was approximately 150 for highly exposed human populations.

Perfluorooctanoic acid (PFOA) is a perfluoroalkyl acid (PFAA) polymerization aid used in the manufacture of fluorinated polymers and elastomers. The resulting fluorinated compounds are used in myriad consumer and industrial products, including nonstick, stain-repellant, water-repellant, and fire-retardant coatings. PFOA is also a known breakdown product of fluorinated telomer alcohols and other precursor compounds of fluorinated polymers (Wang et al. 2005). Production of PFOA worldwide exceeded 1,000 metric tons in 2004, and it is widely distributed and persistent in the environment. The presence of PFOA in serum and tissues of humans and wildlife indicate that exposure to PFOA is widespread.

Levels of PFOA in wildlife range from 0.05 ng/mL in the blood of cod collected from European waters (Falandysz et al. 2006) to 8.14 ng/mL in plasma of loggerhead sea turtles from North America (Keller et al. 2005). Human serum concentrations vary depending on the population evaluated: PFOA concentrations between 4.8 and 5.5 ng/mL were reported in the general U.S. population (Calafat et al. 2007), whereas a population living near a fluoropolymer production facility had values of 386–824 ng/mL for occupational exposures and 307–458 ng/mL for environmental exposures (Emmett et al. 2006). Epidemiologic studies to explore potential human health effects are ongoing (Fletcher et al. 2007).

Liver toxicity is commonly reported in exposed laboratory animals. Hepatomegaly and hepatic peroxisome proliferation have been described in monkeys (Butenhoff et al. 2002) and in various strains of mice (Kudo et al. 2006; Yang et al. 2000, 2001) and rats (Biegel et al. 2001; Kudo et al. 2000; Palazzolo 1993). Liver, Leydig cell (testis), and acinar cell (pancreas) tumors have also been reported in rodents (Biegel et al. 2001; Cook et al. 1999). Immunotoxicity has also been reported (Yang et al. 2000, 2001, 2002). In addition, numerous developmental effects, including neonatal mortality, postnatal growth impairment, developmental delays, and retarded growth, have recently been reported (Lau et al. 2006; White et al. 2007; Wolf et al. 2007).

Lymphoid tissue atrophy (Yang et al. 2000, 2001) and reduced *de novo* antibody synthesis (Yang et al. 2002) have been observed in C57BL/6 mice following dietary exposure to PFOA. Thus, a preliminary risk assessment by the U.S. Environmental Protection Agency (EPA) identified immuno-suppression as an end point of concern; a subsequent review of the risk assessment by the U.S. EPA Science Advisory Board (U.S. EPA 2006) recommended that immune system effects be considered for quantitative risk assessment. The level of U.S. EPA interest and lack of corroborating studies warranted a more thorough assessment. We therefore evaluated both humoral and cell-mediated immune function in experiments designed to corroborate the reported altered immune function observed in C57BL/6 mice and to establish no observed adverse effect level (NOAEL) and lowest observed adverse effect level (LOAEL) values from dose–response studies of immune function.

## Materials and Methods

### Animals

We used the C57BL/6 mouse strain for consistency with the studies of Yang et al. (2000, 2001, 2002). C57BL/6J female mice (6–7 weeks of age) were purchased for the initial (recovery) study from the Jackson Laboratories (Bar Harbor, ME). However, during the course of that study, many of the mice had skin lesions. We later learned that C57BL/6J mice have become genetically susceptible to ulcerative dermatitis. Thus, for the dose–response studies, we purchased C57BL/6N female mice (6–7 weeks of age) from Charles River Laboratories (Raleigh, NC). Once at the U.S. EPA’s animal care facilities (accredited by the Association for Assessment and Accreditation of Laboratory Animal Care), animals were housed in groups of eight in polycarbonate cages with hardwood chip bedding (Beta Chip; Northeastern Products, Warrensburg, NY). They were provided a 12-hr light:dark cycle (light, 0600–1800 hours; dark, 1800–0600 hours), maintained at 22.3 ± 1.1°C and 50 ± 10% humidity, and given *ad libitum* access to both food (5P00 Prolab RMH 3000; PMI Nutrition International, Richmond, IN) and water. Animals were acclimated for at least 10 days before dosing began. All procedures employed in this study were approved in advance by the Institutional Animal Care and Use Committee of the National Health and Environmental Effects Research Laboratory, U.S. EPA; all animals were treated humanely and with regard for alleviation of suffering.

### Recovery study

#### Dosing solutions

**Figure 1:**
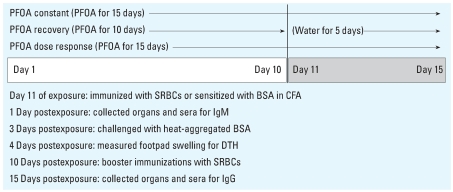
Study design of recovery and dose–response studies.

PFOA was purchased from Fluka Chemical (Steinhiem, Switzerland) as its ammonium salt (≥ 98% purity, lot 421207/1 319030). PFOA dosing solutions were prepared fresh twice weekly in deionized water at a concentration of 3 mg/mL. Vehicle control mice received water vehicle by gavage once daily for 15 days. Experimental groups were exposed to 30 mg PFOA/kg body weight (BW) per day by gavage for 10 days; on days 11–15 of dosing, half of the mice receiving PFOA were switched to the water vehicle (recovery group) and the other half continued receiving PFOA (constant group; Figure 1). We chose the dose of 30 mg/kg/day because Yang et al. (2000, 2001, 2002) reported that this dose reduced lymphoid organ weights and production of antigen-specific antibodies over a similar time period.

#### Experimental design

Animals were randomly divided into 40 animals/end point and 8 animals/dose group. Animals were weighed twice weekly during the dosing period and also just before sacrifice. We conducted cellular and humoral immune function assays in separate groups of animals. Cage controls were included with each end point group to ensure that gavage treatment did not alter experimental results and, with the exception of gavage exposure, were treated identically to all other mice within end point groups.

#### Antibody synthesis (IgM and IgG)

Animals (16/dose) were immunized on the 11th day of dosing by intravenous injection of 4.0 × 10^7^ sheep red blood cells (SRBCs) in 0.2 mL sterile saline. Five days later, 8 animals/ dose were anesthetized with carbon dioxide and exsanguinated by neck vein transection. Blood was collected and held at room temperature for 30 min, centrifuged at 4°C to separate serum, and serum was frozen at –80°C until analysis of SRBC-specific IgM. Two weeks after primary immunization, the remaining 8 animals/dose were given a booster immunization of SRBCs (4.0 × 10^7^). Five days later, animals were anesthetized with carbon dioxide and exsanguinated by neck vein transection. Blood was processed as described above for later analysis of SRBC-specific IgG. The relative serum titers of SRBC-specific IgM and IgG antibodies were measured by ELISA as described below.

IgM titers were determined as described previously (DeWitt et al. 2005). Briefly, flat-bottom 96-well Immunolon-2 ELISA microtiter plates (Dynatech Labs, Chantilly, VA) were coated with 125 μL of 2 μg/mL of SRBC membrane [1.46 mg/mL stock solution diluted in phosphate-buffered saline (PBS); prepared according to Temple et al. (1995)] and then incubated at 4°C for at least 16 hr. Each plate included 20 wells that were coated with pooled serum collected from healthy mice 5 days after primary immunization with SRBCs, and 16 wells contained 100 μL PBS as blanks. After washing, blocking of nonspecific binding, and addition of serum samples (serially diluted from 1:8 to 1:4,096), secondary antibody (goat anti-mouse IgM horseradish peroxidase; Accurate Chemical and Scientific Corp., Westbury, NY) was added. Following three washes and addition of substrate [one tablet of 2,2′-azino-di-(3 ethylbenzthiazoline sulfonic acid) (ABTS; Sigma Chemical Company, St. Louis, MO); added to 50 mL phosphate-citrate buffer with one tablet of urea hydroxide peroxide (Sigma) in 100 mL of distilled water], plates were incubated for 45 min at room temperature and then read at 410 nm on a SpectraMax 350 plate reader (Molecular Devices, Sunnyvale, CA).

The concentration of SRBC-specific IgG was evaluated using the same quantitative assay as for IgM with the following exceptions: The internal control for IgG was generated by serial dilution (from 1:8 to 1:4,096) of 100 μL pooled serum collected from healthy mice 5 days after a second immunization with SRBCs; F(ab′)2 goat anti-mouse IgG horse-radish peroxidase (Accurate Chemical and Scientific Corp., Westbury, NY) was used as the secondary antibody. Absorbance was read on a SpectraMax 350-plate reader at 410 nm. Both IgM and IgG antibody titers were processed using SOFTmax Pro software (Molecular Devices) to determine the log_2_ serum titer for an optical density of 0.5 units from the log–log curve of optical density versus dilution, as described by Temple et al. (1995).

#### Delayed-type hypersensitivity responses (DTH)

Eight animals per dose were used to measure the DTH responses to purified (fraction V) bovine serum albumin (BSA; Sigma). BSA (2 mg/mL in sterile saline) was emulsified in Freund’s complete adjuvant (CFA; Difco, Detroit, MI) at a 1:1 ratio. Animals anesthetized with isoflurane were sensitized on the 11th day of dosing by injecting 0.05 mL BSA-CFA subcutaneously into the caudal tail fold. After 7 days animals were anesthetized with isoflurane and challenged by injecting 0.05 mL of heat-aggregated BSA into the right rear footpad. The left rear footpad was injected with the same volume of saline and served as the injection control. BSA was aggregated by heating 40 mg BSA/mL of sterile saline to 75°C for 1 hr and removing excess saline by centrifuging for 10 min at 450 × *g*. After 24 hr, footpad thickness (triplicate measurements) was determined in anesthetized animals with an electronic caliper designed and built in the model shop at the U.S. EPA (Research Triangle Park, NC). The device applies very light, even, and reproducible pressure on the footpad for each measurement, thus increasing the accuracy of measurements. Standards of known thickness were measured before and after experimental measurements. Swelling was calculated by subtracting the mean saline-injected, left footpad thickness from the mean BSA-injected right footpad thickness.

#### Lymphoid organ weights

Lymphoid organs (spleen and thymus) were removed from animals that were bled for IgM (1 day after exposure ended) and IgG (15 days after exposure ended) for antibody determinations. The organs were immediately weighed and archived at –80°C.

### Dose–response study I

#### Dosing solutions

PFOA drinking water dosing solutions were prepared fresh twice weekly in deionized water at concentrations of 200, 100, 50, and 25 mg/L (to provide doses of 30, 15, 7.5, and 3.75 mg/kg/day, respectively, based on average daily water consumption rates and animal body weights). A 200-mg/L solution was initially prepared by adding PFOA to deionized water and mixing for 5 min. Lower concentrations were made by serial dilutions in deionized water. Dosing solutions were mixed in 25-L polycarbonate carboys and then transferred into individually labeled plastic drinking water bottles topped with double ball-bearing sipper tubes. Actual PFOA concentrations in the dosing solutions were determined by liquid chromatography-tandem mass spectrophotometry (LC-MS/MS) analysis and were 224.0 ± 9.1, 109.8 ± 7.6, 53.8 ± 4.8, and 29.7 ± 2.8 mg PFOA/L for target concentrations of 200, 100, 50, and 25 mg/L, respectively. Time-course sampling of water from the dosing solutions over a 4-day period (the maximum time water bottles remained on cages) revealed no significant changes in PFOA concentrations. Mice received PFOA-containing drinking water for 15 consecutive days (Figure 1). Dosing water was changed and water consumption per cage (based on water bottle weights) was recorded twice weekly. Vehicle controls received deionized water for 15 days. Daily PFOA exposure was calculated based on average water consumption per cage. All procedures for dose–response study I were performed twice, with two different groups of animals. All other procedures were identical to those performed in the recovery study.

### Dose–response study II

#### Dosing solutions

The same procedures employed in dose–response study I were used in dose–response study II, with the exception of dosing solution concentrations; PFOA concentrations were 50, 25, 12.5, and 6.25 mg/L (to provide doses of 7.5, 3.75, 1.88, and 0.94 mg/kg/day, respectively, based on average daily water consumption rates). Vehicle controls received deionized water for 15 days. All dosing procedures for dose–response study II were performed twice, with two different groups of animals. All other procedures were identical to those performed in the recovery study.

### Serum PFOA concentrations

#### Sample preparation

We determined PFOA concentrations in aliquots of serum collected for measurement of IgM and IgG titers. Samples from the recovery study and from dose–response study I were prepared in a fashion similar to that of Lau et al. (2006). In brief, serum samples were thawed by placement in cool water and vortexed 30 sec before withdrawing an aliquot (10–25 μL). The aliquot was then placed into a 15-mL polypropylene tube (Falcon tube; BD, Franklin Lakes, NJ).

#### Serum from control animals

Serum was deprotonated with 200 μL 0.1 M formic acid. Samples were then vigorously vortexed for 30 sec and spiked with a 2-mL aliquot of cold acetonitrile containing 1.25 ng ^13^C_2_-PFOA to precipitate the proteins. Samples were centrifuged at 5,000 rpm for 5 min to pelletize proteins, and the supernatant was decanted to a new tube and diluted with 20 mL of fresh deionized water. Samples were concentrated and cleaned up using a Waters (Milford, MA) HLB SPE (hydrophilic-lipophilic balance, solid-phase extraction) column (3 cc, 60 mg) with a water wash and a methanol extraction. Methanolic extracts were then reduced under N_2_ gas to an appropriate volume using a Zymark N-Evap (Organomation Associates Inc., Berlin, MA). The analytes were redissolved in equal volume of 2 mM ammonium acetate and acetonitrile for LC/MS-MS analysis.

#### Serum from dosed animals

Serum was diluted and deprotonated with 10 mL of 0.1 M formic acid. Samples were shaken for 1 hr; a 1-mL aliquot was then removed and diluted with cold acetonitrile containing 100 ng ^13^C_2_-PFOA. After shaking (30 min), a 200-μL aliquot was removed and combined with 2 mM ammonium acetate for LC/MS-MS analysis.

#### Standard curve and quality assurance/ quality control (QA/QC)

The standard curve preparation was matrix matched. Standards were prepared by placing 10–25 μL of control Pel-Freez CD1 mouse serum (Pel-Freez Biologicals, Rogers, AR) in a 15-mL polypropylene tube and adding a known mass of PFOA in methanol relating to 1–200 ng PFOA/mL serum for controls and 10,000–300,000 ng PFOA/mL serum for dosed animals. QA/QC samples were prepared in advance in a batch of Pel-Freez CD1 mouse serum at 25 and 100 ng/mL for control animal sera and 25,000 and 100,000 ng/mL for dosed animal sera. The average accuracy was 104 ± 14.4% and 89.4 ± 11.0% for low and high (control) QC sera, respectively, and 104 ± 11.1% and 98.0 ± 10.2% for low and high (dosed) sera, respectively. Average coefficients of variation for replicate analysis of unknown (*n* = 20) sera for both control and dosed animals was 11.7%.

Samples were run in batches that included double blanks (solvent blank), a method blank, matrix blank (blank serum), standards, QC samples, and unknowns in sequence. Standards were run at the beginning and end of the analytical batch, and QC samples were interspersed in the analytical batch. PFOA was monitored via the transition 413-369 and for the ^13^C-PFOA 415-370. Samples were run using an isocratic mobile phase of 30:70 2 mM ammonium acetate:acetonitrile at a flow of 200 μL/min with a 10-μL sample volume. Samples were run through a Sunfire C18 column (50 × 3.0 mm, 5 μm particle size; Waters) and appropriate guard column for separation. Analytes were integrated using the equipment software and corrected if necessary by the operator. Quantitation was done by making standard curves of concentration versus area ratios of internal standard response to analyte response. All standard curves had *r*^2^ values > 0.99.

#### PFOA dosing solutions

Dosing solutions of PFOA dissolved in deionized water were analyzed from animal-watering apparatus to ensure no appreciable losses of PFOA occurred between changing solutions. Dosing solutions were diluted to bring PFOA concentrations into an acceptable range for LC-MS/MS analysis with deionized water and analyzed as above by LC-MS/MS. Concentrations of PFOA in dosing solutions did not change appreciably over a 2-week period (data not shown).

### Benchmark dose analysis

We pooled IgM serum titer data from two replicate experiments that identified both a LOAEL and NOAEL and analyzed them to determine the benchmark dose (BMD) and lower bound of the 95% confidence interval, using software developed by the U.S. EPA (2000). One standard deviation (1SD) from the control mean was chosen as the benchmark response (BMD_1SD_) level. The current BMD technical guidelines (U.S. EPA 2000) suggest 1SD from the control mean as a benchmark response level for continuous data in the absence of additional information such as a minimal level of change in the end point that is generally considered to be biologically significant. Effects on serum titer were modeled using Hill, power, and first, second, and third degree polynomial models for homogeneous data.

### Statistical analysis

All data are presented as mean ± SE. Statistical analyses were performed with the SAS System (SAS Institute, Cary, NC). We used analysis of variance (ANOVA) to analyze immune responses by dose; when appropriate, linear regressions were used to determine dose response. When ANOVA indicated a statistically significant treatment effect, we made individual post hoc comparisons using Tukey’s test and the least squares means *t*-test with a Tukey’s adjustment for controlling the family-wise error rate. A repeated-measures ANOVA was used to analyze BW changes over time and dose. Statistical significance was determined using an α of 0.05.

## Results

### Recovery study

#### Serum PFOA concentrations

In the recovery and constant groups, respectively, the fold difference in mean PFOA concentration relative to vehicle controls was 2,582 and 8,125 1 day after dosing ended, and 1,933 and 2,753 15 days after dosing ended (Table 1). Mean differences in PFOA concentration between vehicle and cage controls did not differ statistically, although the mean value for the cage controls 15 days after exposure ended (614.9 ng PFOA/mL) indicated a source of PFOA contamination for this group of animals. However, levels of PFOA in all plastic materials used during the experiment were at or below the limit of detection.

#### Water consumption and body and liver weights

**Figure 2:**
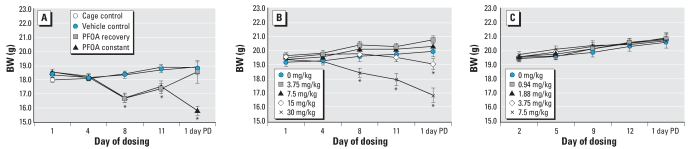
Effects of PFOA exposure on BW (mean ± SE) of female C57BL/6J (*A*) or C57BL/6N (*B*,*C*) mice. PD, postdosing. (*A*) Effects of PFOA administered via gavage to C57BL/6J mice for 10 days (PFOA-recovery group) or 15 days (PFOA-constant group). Mean BWs of both the recovery and constant groups were reduced compared with vehicle controls from the 8th–11th days of dosing; however, by the 12th day of dosing, BWs of the recovery group had returned to control levels. (*B, C*) Effects of various concentrations of PFOA, given for 15 days via drinking water, on mean BW in female C57BL/6N mice. (*B*) Mean BW of the 30-mg PFOA/kg dose group was reduced compared with vehicle controls beginning the 8th day of dosing; mean BW of the 15-mg PFOA/kg dose group was reduced relative to vehicle controls 1 day after dosing ended. (*C*) Mean BWs of mice exposed to 0.94–7.5 mg PFOA/kg did not change by dose within the 15-day exposure period. *p < 0.05.

Water consumption per cage did not vary statistically between vehicle controls and dosed groups (data not shown). From the 8th day of dosing through the 11th day of dosing, mean BW of animals receiving 30 mg PFOA/kg/day (recovery and constant) was reduced by approximately 8% (*p* < 0.05) relative to BW of animals receiving vehicle (Figure 2A). Two days after PFOA dosing ended, the mean BW of animals from the recovery group was equivalent to the weights of the vehicle control group. Weights of the constant group remained reduced by approximately 10.5% relative to the weights of the vehicle control group until the end of dosing (*p* < 0.05). Fifteen days after exposure ended, the BW of animals from all groups were statistically equivalent (data not shown). Relative liver weights for both the constant and recovery groups were elevated (*p* < 0.05) by 64% compared with controls 1 day after exposure ended and by approximately 54% 15 days after exposure ended (data not shown).

#### Lymphoid organ weights

One day after dosing ended, mean spleen and thymus weights were reduced in animals exposed to 30 mg PFOA/kg compared with unexposed animals (Table 2). Absolute and relative spleen weights were approximately 24% lower in the recovery group and approximately 48% lower in the constant group (*p* < 0.05). Absolute and relative thymus weights were approximately 36% lower in the recovery group and approximately 79% lower in the constant group (*p* < 0.05). By 15 days after the end of exposure, both spleen and thymus weights were statistically equivalent to control weights.

#### Immune responses

**Figure 3:**
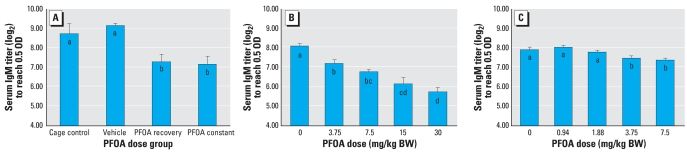
Effects of PFOA exposure on SRBC-specific IgM antibody titers (mean ± SE) in female C57BL/6J (*A*) or C57BL/6N (*B*,*C*) mice. OD, optical density. (*A*) PFOA was given for 10 days (PFOA recovery) or 15 days (PFOA constant) via gavage; IgM antibody titers were suppressed in both exposed groups compared with controls. (*B*,*C*) PFOA was given for 15 days via drinking water. (*B*) IgM antibody titers were suppressed relative to controls for all tested doses. (*C*) IgM antibody titers were suppressed relative to controls at the two highest doses. Means with different letters are statistically different (*p* < 0.05).

**Figure 4:**
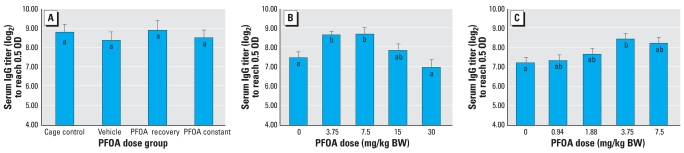
Effects of PFOA exposure on SRBC-specific IgG antibody titers (mean ± SE) in female C57BL/6J (*A*) or C57BL/6N (*B*,*C*) mice. OD, optical density. (*A*) PFOA (30 mg/kg) was given for 10 days (PFOA recovery) or 15 days (PFOA constant) via gavage. No statistical effect on IgG antibody titers was detected. (*B*, *C*) PFOA given for 15 days via drinking water. (*B*) IgG antibody titers were elevated compared with controls at 3.75 and 7.5 mg/kg. (*C*) IgG antibody titers were elevated compared with controls at 3.75 mg/kg. Means with different letters are statistically different (*p* < 0.05)

SRBC-specific IgM antibody titers (Figure 3A) were reduced (*p* < 0.05) relative to controls by nearly 20% in both the recovery and the constant groups. SRBC-specific IgG antibody titers (Figure 4A) and DTH responses (data not shown) were not statistically altered by exposure to PFOA.

### Dose–response study I

#### Serum PFOA concentrations

One day after dosing ended, the mean PFOA concentration of drinking water was between 7.5 × 10^4^ (3.75 mg PFOA/kg/day) and 1.6 × 10^5^ ng/mL (30 mg PFOA/kg/day) (Table 1). Linear regression analysis indicated that PFOA concentrations among the dose groups increased dose dependently (*r*^2^ = 0.7711). Fifteen days after dosing ended, the mean PFOA concentrations had decreased to 3.5 × 10^4^ ng PFOA/mL in the 3.75-mg PFOA/kg/day dose group and 5.3 × 10^4^ ng PFOA/mL in the 30-mg PFOA/kg/day dose group. Within 2 weeks, the mean PFOA concentration was approximately 50% lower relative to the 1-day postdosing concentrations in the two low dose groups (3.75 and 7.5 mg PFOA/kg/day) and approximately 65% lower in the two high dose groups (15 and 30 mg PFOA/ kg/day). The mean PFOA concentrations in the vehicle control groups were between 54.3 ng PFOA/mL 1 day postdosing and 156.4 ng PFOA/mL 15 days postdosing. The 15-day postdosing PFOA vehicle control concentration indicated a source of PFOA contamination or transfer of PFOA from another cage; however, as with the cage controls in the recovery study, levels of PFOA in all plastic materials used during the experiment were at or below the limit of detection on the instrument.

#### Water consumption and body and liver weights

Water consumption per cage did not vary statistically between vehicle controls and dosed groups (data not shown). From the 8th day of dosing through the end of the dosing period, mean BW (Figure 2A) of animals drinking 30 mg PFOA/kg/day was reduced by 6–15% (*p* < 0.05) relative to BW of animals drinking vehicle water. The BW of animals drinking 15 mg PFOA/kg/day was reduced by nearly 6% at the end of the 15-day dosing period. BWs of animals drinking 30 mg PFOA/kg were equivalent to those of control animals within 8 days after dosing ended (data not shown). Relative liver weights for all dosed groups were elevated (*p* < 0.05) compared with controls by 51–70% 1 day after exposure ended and 45–61% 15 days after exposure ended (data not shown).

#### Lymphoid organ weights

One day after exposure ended, mean spleen and thymus weights of animals exposed to 15 or 30 mg PFOA/kg/day were reduced relative to unexposed animals (Table 2). Absolute and relative spleen weights were approximately 32% lower in the 15-mg PFOA/kg/day group and 44% lower in the 30-mg PFOA/kg/day group (*p* < 0.05). Absolute and relative thymus weights were approximately 31% lower in the 15-mg PFOA/kg/day group and 52% lower in the 30-mg PFOA/kg/day group (*p* < 0.05). We observed no changes to lymphoid organ weights at any of the lower doses. Fifteen days postdosing, both spleen and thymus weights were statistically equivalent to control weights. The exception was the 15-mg PFOA/kg/day group, in which the mean absolute thymus weight was 18% greater (*p* < 0.05) relative to control weights.

#### Immune responses

All doses of PFOA reduced SRBC-specific IgM antibody titers (Figure 3B) relative to controls (*p* < 0.05) by between 11 (3.75 mg PFOA/kg/day) and 29% (30 mg PFOA/kg/day). Therefore, a NOAEL for suppressed IgM antibody production was not identified with this study. We identified 3.75 mg PFOA/kg/day as the LOAEL for suppressed IgM antibody production. SRBC-specific IgG antibody titers (Figure 4B) were not reduced relative to controls at any dose tested; however, titers were elevated relative to controls by approximately 13% in both the 3.75- and 7.5-mg PFOA/kg/day groups. DTH responses (data not shown) were not statistically altered by exposure to the tested doses of PFOA.

### Dose–response study II

#### Water consumption and body and liver weights

Water consumption per cage did not vary statistically between vehicle controls and dosed groups (data not shown). BWs of animals exposed to 0.94–7.5 mg PFOA/kg/day did not statistically differ from BW of control animals during the dosing period (*p* < 0.05; Figure 2B). Relative liver weights for all dosed groups were elevated (*p* < 0.05) relative to controls by 35–60% 1 day after exposure ended and 22–45% 15 days after exposure ended (data not shown).

#### Lymphoid organ weights

One day after exposure ended, mean spleen weights of animals exposed to 3.75 or 7.5 mg PFOA/kg/day were reduced relative to unexposed animals (Table 2). Absolute and relative spleen weights were approximately 16% lower in the 3.75-mg PFOA/kg/day group and 18% lower in the 7.5-mg PFOA/kg/day group (*p* < 0.05). Fifteen days postdosing, spleen weights of these groups were statistically equivalent to control weights. No other statistical changes in lymphoid organ weights were observed.

#### Immune responses

Exposure to 3.75 or 7.5 mg PFOA/kg/day reduced SRBC-specific IgM antibody titers (Figure 3C) by approximately 7% relative to controls (*p* < 0.05). We identified a NOAEL of 1.88 mg PFOA/kg/day for suppressed IgM antibody production; these results supported the LOAEL of 3.75 mg PFOA/kg/day from dose–response study I. SRBC-specific IgG antibody titers (Figure 4C) were elevated by 14% relative to controls in the 3.75 mg PFOA/kg/day group. DTH responses (data not shown) were not statistically altered by exposure to the tested doses of PFOA.

### BMD analysis

All models properly described the response and detected significant differences between dose levels. Although the lowest Akaike information criterion (AIC) number and the smallest scaled residual value at the BMD were returned by the Hill model, the model failed to detect a lower bound to the BMD, perhaps because the selected benchmark response fell outside the range of possible asymptote values. Results of the second-degree polynomial model were therefore chosen as the best representation of the data, with an AIC of –59.10 and a scaled residual value of 0.24. The model returned a BMD_1SD_ of 3.06 mg/kg/day and a lower 95% confidence limit of 1.75 mg/kg/day.

## Discussion

In this study, we evaluated lymphoid organ weights and two adaptive immune responses in adult C57BL/6J or C57BL/6N mice following exposure to the PFOA, a PFAA. Exposure to 15 or 30 mg PFOA/kg/day after 10 or 15 days of exposure (via gavage or drinking water) statistically reduced relative spleen and thymus weights and SRBC-specific IgM antibody titers. In addition, 3.75 and 7.5 mg PFOA/kg/day also reduced the production of SRBC-specific IgM antibodies when given via drinking water for 15 days. This is in contrast to the results of Yang et al. (2002), who reported that the IgM response was suppressed only by constant exposure to PFOA. Our data therefore suggest that in rodents, adaptive immune functions may be sensitive to PFOA at concentrations ≥ 3.75 mg/kg/day, a dose that resulted in a serum concentration of 7.5 × 10^4^ ng/mL. This concentration is approximately 50- to 100-fold greater than the concentration of PFOA reported in sera of humans living near a PFOA production facility.

Within the past decade, considerable attention has been paid to PFOA and related PFAAs because of their presence in humans, biota, and environmental media. However, the toxicokinetics and mode(s)/mechanism(s) of action for toxicologic effects have not yet been thoroughly described. Although immuno-suppression has been identified as an end point of concern by the U.S. EPA, a paucity of data exists to corroborate the few studies that report immune suppression after exposure to PFOA.

The changes that we observed in lymphoid organ weights for our recovery study and in dose–response study I were consistent with previous reports of PFOA-induced immuno-modulation (Yang et al. 2000, 2001). Both laboratories observed reductions in lymphoid organ weights at 15 and 30 mg PFOA/kg/day, but not at lower doses, and also demonstrated that lymphoid organ weights recovered to control levels within 10 (Yang et al. 2001) or 15 days after exposure ended. At doses of 15 and 30 mg PFOA/kg/day, mean serum PFOA concentrations for our animals were between 5.0 × 10^4^ ng/mL (dose–response study I) and 6.8 × 10^4^ ng/mL (recovery study) 15 days after exposure ended, indicating that circulating PFOA at this concentration was not sufficient to maintain suppressed lymphoid organ weights.

The data from our dose–response study I indicated that reductions in IgM antibody titers occurred at lower doses and PFOA serum concentrations than those required to reduce lymphoid organ weights. In dose–response study I, lymphoid organ weight reductions occurred only at doses of 15 and 30 mg PFOA/kg/day, corresponding to mean PFOA serum concentrations of 1.3 × 10^5^ and 1.6 × 10^5^ ng/mL, respectively. IgM antibody titers were reduced at a dose of 3.75 mg PFOA/kg/day and a PFOA serum concentration of 7.5 × 10^4^ ng/mL, which we identified as a LOAEL. Therefore, because statistical reductions in lymphoid organ weights did not coincide with effects on antibody synthesis, we conclude that lymphoid organ atrophy is not a sensitive indicator of PFOA-induced immune dysfunction.

In both dose–response studies, we identified a LOAEL of 3.75 mg PFOA/kg/day, and in dose–response study II, we identified a NOAEL of 1.88 mg PFOA/kg/day for IgM antibody responses. Although Yang et al. (2002) evaluated the effects of PFOA on several immune system parameters, they used only one dose (30 mg PFOA/kg/day). At this PFOA dose, Yang et al. (2002) reported suppression of horse red blood cell (HRBC)–specific IgM and IgG antibody titers, a reduction in the number of spleen cells secreting HRBC-specific IgM and IgG antibodies, and a reduction in the proliferative response of spleen cells after stimulation; however, methodologic discrepancies make interpretation of their data difficult. For example, Yang et al. (2002) reported HRBC-specific plaques and IgM/IgG antibody titers in unimmunized animals and measured IgG after only a single immunization. These discrepancies suggest that the magnitude of the suppression due to PFOA exposure may differ from what was reported by Yang et al. (2002). Our data support suppression of antibody titers at a dose of 30 mg/kg/day (Yang et al. 2002) and indicate that suppression of the primary antibody response occurs at a dose that is approximately 8-fold lower than reported by Yang et al. (2002). Application of BMD analysis of pooled low-dose datasets (0, 0.94, 1.88, 3.75, and 7.5 mg/kg/day) returned a BMD of 3.06 mg PFOA/kg/day, with a lower bound of 1.75 mg PFOA/kg/day. The lower bound value is selected as the point of departure by risk assessors, and is used to set reference doses (RfD) or concentrations. An advantage of the BMD approach is that all data from the dose response are used, and the derived dose does not depend on investigator-selected dose intervals used to identify LOAELs and NOAELs. Values derived by all BMD models were within 1–2 mg PFOA/kg/day, suggesting that a dose in this range is a reasonable point of departure for calculating an immunotoxicity-based RfD.

We also determined that IgG responses were affected by PFOA exposure, although in our studies, IgG titers were increased at lower doses and were similar to control responses at the higher doses. These results are in contrast to suppression of the IgG response reported by Yang et al. (2002) and by Cunard et al. (2002), who reported that exposure to the potent peroxisome proliferator-activated receptor α (PPAR-α) agonist and the peroxisome proliferator WY14,643 suppressed IgG antibody responses and reduced splenocyte number. However, our results may reflect recovery and rebound of IgG synthesis at lower doses, and progressive—but not complete—recovery from PFOA-induced suppression at higher exposure levels. Because our data reflect function approximately 2 weeks after exposure ended, additional studies will be conducted in which the booster immunization is delayed; this modification may help to resolve the apparent augmented IgG response in exposed animals. In addition, the antibody titer and plaque-forming cell results reported by Yang et al. (2002) do not reflect IgG levels collected at peak response or following booster immunizations; they collected IgG data after only a single immunization and at the same time as IgM data.

Serum levels of PFOA in the general human population have been reported to be approximately 5 ng/mL (Calafat et al. 2007) and approximately 5 × 10^2^ ng/mL (Emmett et al. 2006) after environmental/occupational exposures. Using our LOAEL of 3.75 mg PFOA/kg/day for suppressed IgM antibody production and the associated serum PFOA concentration of 7.5 × 10^4^ ng/mL, the margin of exposure for suppressed IgM antibody production is approximately 150 for environmental/occupational exposures and 15,000 for the general human population. However, we observed changes in IgG antibody titers at a serum PFOA concentration as low as 3.5 × 10^4^ ng/mL, which is a margin of exposure of only 70 for environmentally/occupationally exposed populations. Our antibody data and the data reported by Yang et al. (2000, 2001, 2002) suggest that the immune system is a target of PFOA and that the recommendation by the U.S. EPA Science Advisory Board (U.S. EPA 2006) to consider immune system effects for a quantitative PFOA risk assessment is warranted.

We are currently exploring mechanisms by which PFOA may reduce IgM antibody titers at a lower dose than that at which significant effects on lymphoid organ weights are observed, as well as the relationship between PFOA-induced peroxisome proliferation and immunomodulation in C57BL/6 mice. Peroxisome proliferation, as evidenced by the liver hepatomegaly that we observed at all tested doses, occurred at lower doses than required to reduce lymphoid organ weights or antibody production. We are evaluating immune responses in C57BL/6 mice lacking PPAR-α, a receptor that is activated by PFOA, to determine the role of PFOA-induced PPAR-α activation in immunomodulation.

## Figures and Tables

**Table 1:** Serum concentrations (mean ± SE) of PFOA from female mice exposed for 15 days, with serum collected 1 or 15 days postdosing (PD).

	1 Day PD (ng/mL)	15 Days PD (ng/mL)
Recovery study^a^		
Cage control	25.2 ± 2.0*a	614.9 ± 66.6a
Vehicle control	32.8 ± 19.5a	24.7 ± 2.0a
Recovery	84,700 ± 9,814b	47,757 ± 2,115b
Constant	266,500 ± 23,018c	67,988 ± 3,853c
Dose–response study^b^		
0 mg/kg	54.3 ± 4.9a	156.4 ± 14.9a
3.75 mg/kg	74,913 ± 2,667b	35,325 ± 1,607b
7.5 mg/kg	87,150 ± 3,296b,c	42,771 ± 1,708b
15 mg/kg	128,125 ± 6,818c	50,025 ± 1,486b,c
30 mg/kg	162,625 ± 8,434d	52,713 ± 3,212c

a C57BL/6J mice were treated with PFOA by gavage; serum from recovery groups was collected 6 (1 day PD) or 20 (15 days PD) days after the last PFOA dose was administered.

b C57BL/6N mice were treated with PFOA in drinking water.

* Within 1-day or 15-days PD groups, means followed by a different letter are statistically different (*p* < 0.05).

**Table 2:** Lymphoid organ weights (mean ± SE) of female mice exposed to PFOA for 15 days, 1 or 15 days postdosing (PD).

	Spleen weight (mg)		Relative spleen weight		Thymus weight (mg)		Relative thymus weight	
Recovery study^a^	1 day PD	15 days PD	1 day PD	15 days PD	1 day PD	15 days PD	1 day PD	15 days PD
Cage control	91.9 ± 7.62	89.5 ± 4.12	4.84 ± 0.32	4.55 ± 0.20	49.1 ± 2.06	50.1 ± 1.89	2.61 ± 0.078	2.55 ± 0.075
Vehicle control	90.6 ± 2.13	82.6 ± 4.50	4.80 ± 0.11	4.36 ± 0.19	49.4 ± 1.36	20.1 ± 1.97	2.62 ± 0.071	2.66 ± 0.10
Recovery	68.2 ± 5.13*	82.9 ± 3.76	3.72 ± 0.37*	4.16 ± 0.13	31.3 ± 7.42*	56.3 ± 4.74	1.69 ± 0.41*	2.83 ± 0.22
Constant	43.8 ± 2.28*	72.8 ± 5.18	2.77 ± 0.12*	3.72 ± 0.22	9.6 ± 1.41*	43.4 ± 5.34	0.60 ± 0.081*	2.22 ± 0.28
Dose–response study I (PFOA mg/kg/day)								
0	89.9 ± 2.59	86.3 ± 2.86	4.63 ± 0.17	4.16 ± 0.13	62.1 ± 2.60	61.3 ± 2.44	3.19 ± 0.14	2.97 ± 0.14
3.75	93.9 ± 12.12	95.2 ± 1.73	4.57 ± 0.55	4.43 ± 0.084	62.9 ± 2.44	67.3 ± 2.29	3.09 ± 0.13	3.13 ± 0.11
7.5	78.5 ± 2.35	92.2 ± 2.24	3.80 ± 0.12	4.35 ± 0.078	59.0 ± 3.14	64.0 ± 2.17	2.88 ± 0.18	3.01 ± 0.077
15	60.8 ± 1.77*	95.3 ± 2.29	3.18 ± 0.068*	4.43 ± 0.078	42.7 ± 2.94*	72.3 ± 1.49*	2.24 ± 0.15*	3.37 ± 0.080
30	47.5 ± 2.62*	79.3 ± 4.19	2.76 ± 0.12*	3.68 ± 0.17	28.2 ± 3.68*	69.3 ± 3.04	1.62 ± 0.21*	3.23 ± 0.14
Dose–response study II (PFOA mg/kg/day)								
0	102.1 ± 4.37	95.1 ± 2.94	4.9 ± 0.18	4.43 ± 0.11	63.1 ± 2.74	58.0 ± 3.74	3.02 ± 0.13	2.69 ± 0.16
0.94	102.1 ± 3.72	99.9 ± 3.12	4.9 ± 0.14	4.65 ± 0.11	64.9 ± 1.32	65.2 ± 2.27	3.16 ± 0.07	3.04 ± 0.096
1.88	94.6 ± 3.42	94.7 ± 4.29	4.6 ± 0.15	4.36 ± 0.20	68.7 ± 2.18	66.3 ± 2.24	3.32 ± 0.10	3.07 ± 0.12
3.75	85.8 ± 2.56*	100.7 ± 2.01	4.1 ± 0.11*	4.62 ± 0.086	58.5 ± 1.71	64.9 ± 2.64	2.80 ± 0.08	2.98 ± 0.12
7.5	82.8 ± 3.32*	103.0 ± 2.73	4.1 ± 0.15*	4.69 ± 0.098	56.9 ± 2.44	67.3 ± 2.20	2.81 ± 0.13	3.08 ± 0.11

a Organs from recovery groups were collected 6 or 20 days after the last PFOA dose was administered.

* Statistically different from control groups (*p*< 0.05).
